# A Review of Solid-State LiDAR Principles and Metasurface-Based LiDAR Sensors

**DOI:** 10.3390/s26010001

**Published:** 2025-12-19

**Authors:** Elif Demirbas, Braden Boucher, Matthew Baker, Joshua Andrews, William Cruz, Sara Mueller, Samuel Serna-Otalvaro

**Affiliations:** Department of Physics, Photonics and Optical Engineering, Bridgewater State University, Bridgewater, MA 02324, USA

**Keywords:** LiDAR, beam steering, phase shift, deflection angle, field of view, metasurfaces, tunable metasurfaces, metasurface-based LiDAR

## Abstract

Light Detecting and Ranging (LiDAR) has been a promising solution for autonomous vehicles. For beam-steering mechanisms, solid-state LiDAR with microelectromechanical systems (MEMS) and optical phased arrays (OPAs) have demonstrated robust and compact alternatives to mechanical LiDAR with 360° rotating mirrors. Two-dimensional optical metasurfaces can be used for phase shift, deflecting the angle in LiDAR. If a LiDAR system only needs a fixed beam direction, then static metasurfaces can be used. If a LiDAR system requires beam scanning, dynamic (tunable) metasurfaces are necessary for efficient and adaptable operation. In this review article, we will discuss the principles of metasurface beam-steering mechanisms and discuss how metasurfaces can shift the incoming light’s phase and deflect the angle. LiDAR based on metasurfaces provides promising solutions due to its flat optics feature, robust nature, and non-moving parts. Additionally, we will discuss and compare the field of view (FOV) of LiDAR based on metasurfaces. Tunable metasurfaces in LiDAR systems are crucial for real-time beam scanning, and they have advantages over traditional mechanical scanning mechanisms like faster scanning rates, increased reliability, more compact form factors, and larger fields of view.

## 1. Introduction

**Figure 1:**
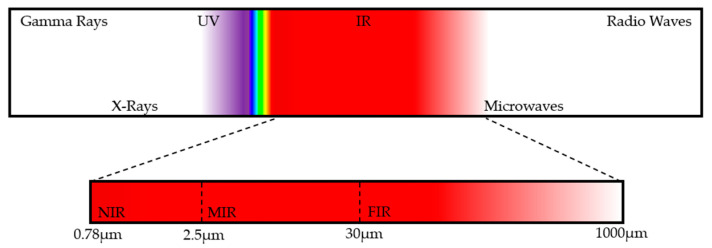
Electromagnetic spectrum showing infrared region inset from 0.78 μm to 1000 μm.

Light Detection and Ranging, or LiDAR for short, is a sensing method that uses light in the near-infrared range to detect and measure distances to an object or obstruction. Near-infrared (NIR) describes the part of the electromagnetic spectrum that ranges from about 0.78 μm to about 2.5 μm [1], which corresponds to a frequency range of about 385 THz to 120 THz. This differs from the more popular term “radar”, which is a sensing and detection method that uses electromagnetic waves in the radio part of the electromagnetic spectrum. This range is much higher in wavelength and describes the part of the spectrum from about 1 mm to 100 km, which corresponds to a frequency range of about 300 GHz to 3 kHz. A diagram of the EM spectrum can be seen in Figure 1. LiDAR uses short laser pulses of NIR light and measures the response of the light after it reflects off an obstruction. The response encodes all sorts of information, such as how far the obstruction is, and the velocity at which the obstruction is moving, if it is at all.

LiDAR has a wide array of applications due to its ability to create highly accurate 3D models of the environment. Some of the key applications are mapping and surveying, environmental monitoring, agriculture, manufacturing, robotics, security and surveillance, military and defense, space, and autonomous vehicles [2]. A popular use of LiDAR is for surveying and mapping to create highly accurate terrain models for urban planning, forestry, and natural resource management [3,4,5]. LiDAR is also used in meteorology to measure atmospheric conditions such as wind speed and direction, temperature, and humidity [6,7]. LiDAR is used in manufacturing for quality control, 3D scanning, and creating a digital version of manufacturing processes [8,9,10]. LiDAR enables robots to navigate, map their surroundings, and interact with objects in a dynamic environment [11,12,13]. LiDAR has been used in missile-deferring systems as well as for tracking and detecting targets in defense applications [14,15,16]. LiDAR has been widely used by NASA to map the surface of planets topographically without landing on the planets [17,18]. The most commercial and popular use of LiDAR currently is in autonomous vehicles [19]. This detection technique allows self-driving cars to map the surrounding environment to detect obstacles, pedestrians, and other vehicles.

The first LiDAR system used in self-driving cars is mechanical LiDAR, which consists of three main components: (1) laser, (2) beam-steering mirror, and (3) detector. The beam-steering mirror mechanically rotates 360° and is very bulky in nature, which limits the location it can be put on the car. In addition to the size, the cost of this LiDAR system is very high. This drives researchers to redesign the LiDAR system to reduce the size and cost. Solid-state LiDAR can be a promising alternative to mechanical LiDAR due to its stationary parts for the beam-steering mechanism.

In this review article, we will introduce the principles of LiDAR sensing schemes like pulsed time of flight (TOF), amplitude-modulated continuous-wave TOF, frequency-modulated continuous-wave, and flash LiDAR. We will discuss the laser types used in each scheme. Later, we will present the LiDAR scanning platforms, such as mechanical LiDAR, and solid-state scanning platforms like microelectromechanical systems (MEMSs) and optical phased arrays (OPAs). Additionally, we will demonstrate the differences between passive and tunable metasurfaces and survey the details of passive metasurfaces applications and tuning mechanisms of active metasurfaces. Lastly, we will show the use of metasurfaces in solid-state LiDAR scanning platforms and review the literature for metasurface-based LiDAR and discuss the use of beam scanning properties like phase shift, deflection angle, and field of view (FOV).

## 2. LiDAR Principles

**Figure 2:**
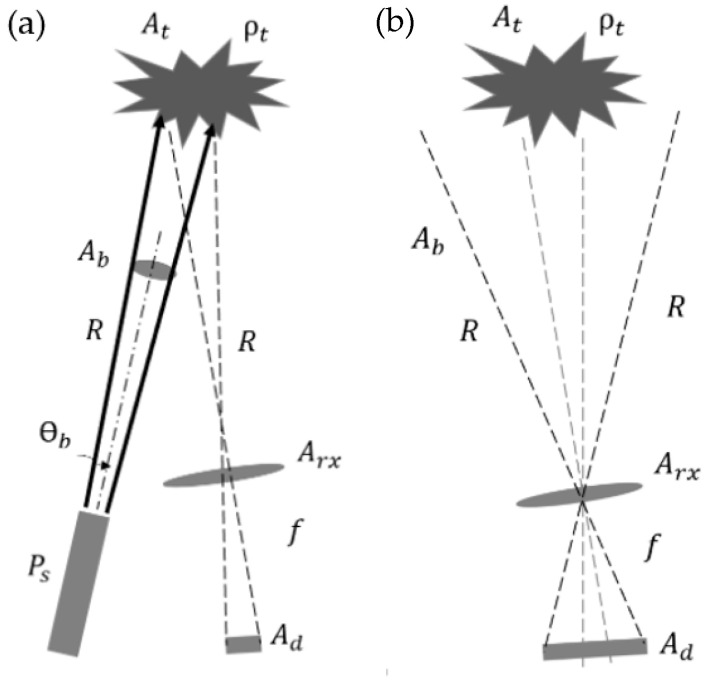
Schematic of hard Lambertian object. (**a**) Receiver FOV-filled: source signal reflects back from hard Lambertian object and illuminates the entire area of receiver. (**b**) Receiver FOV-underfilled: the acceptance range of the receiver is larger than the reflected signal.

The LiDAR signal at the receiver can be calculated as follows by assuming the target as a hard-Lambertian object. A Lambertian surface [20] refers to a physical surface that reflects the light equally in all directions regardless of the viewing angle. There are two schemes (shown in Figure 2): (a) the receiver FOV-filled when the entire acceptance range of receiver is illuminated by the incoming signal; and (b) the receiver FOV-underfilled when the receiver’s area is larger than the incoming signal and not covered entirely.

In receiver FOV-filled LiDAR, to calculate the received LiDAR signal at the receiver, we will show how the source power is transmitted to the target and reflected from the target to the receiver. Source irradiance [W/m^2^] at a target, $E_{t}$, can be written as follows:

(1) $$E_{t}=\frac{P_{s}}{A_{b}}T_{a}=\frac{P_{s}}{\pi {\left(R\theta_{b}\right)}^{2}}T_{a}$$

where $P_{s}$ is the source power; $A_{b}$ is the beam area; $T_{a}$ is the one-way atmospheric transmission; R is the range; and $\theta_{b}$ is the beam angle. The incoming beam is reflected from the target with radiance $L_{r}$ as follows:

(2) $$L_{r}=\frac{\rho_{t}E_{t}}{\pi }=\frac{\rho_{t}P_{s}}{\pi^{2}{\left(R\theta_{b}\right)}^{2}}T_{a}$$

where $\rho_{t}$ is the Lambertian target reflectance. A reflected beam travels through the atmosphere and is received by the detector. The detected power $P_{d}$ can be obtained by multiplying the reflected radiance by the receiver area and atmospheric transmission as follows:

(3) $$P_{d}=L_{r}{\left(A\Omega \right)}_{rx}T_{a}T_{o}≅L_{r}\left(A_{rx}\frac{A_{d}}{f^{2}}\right){T_{a}}^{2}T_{o}=\frac{\rho_{t}P_{s}A_{rx}A_{d}}{\pi^{2}{\left(R\theta_{b}\right)}^{2}f^{2}}{T_{a}}^{2}T_{o}$$

where $A_{rx}$ is the receiver’s aperture area; $A_{d}$ is the detector’s cross-sectional surface area; f is the focal length of the receiver optics; and $T_{o}$ is the optical transmission of the receiver. The receiver’s solid angle ${\left(\Omega \right)}_{rx}$ can be written as $\frac{A_{d}}{f^{2}}\;$ in the receiver FOV-filled case. Using $A_{rx}=\frac{\pi {D_{rx}}^{2}}{4}$ and $F-number=\frac{f}{D_{rx}}$, where $D_{rx}$ is the diameter of the detector area, the detected power can be written as a function of R and $\theta_{b}$:

(4) $$P_{d}\left(R,\theta_{b}\right)=\frac{\rho_{t}P_{s}A_{d}}{{4\pi }^{2}{\left(R\theta_{b}\right)}^{2}F^{2}}{T_{a}}^{2}T_{o}$$

where the *F − number* of detectors plays a key role in the detected LiDAR signal.

When the receiver FOV is under-filled, the receiver’s solid angle ${\left(\Omega \right)}_{rx}$ becomes $\frac{A_{t}}{R^{2}}$ where $A_{t}$ is the target area. In this case, the detected power is as follows:

(5) $$P_{d}=L_{r}{\left(A\Omega \right)}_{rx}T_{a}T_{o}≅L_{r}\left(A_{rx}\frac{A_{t}}{R^{2}}\right)T_{a}T_{o}=\frac{\rho_{t}P_{s}A_{rx}A_{t}}{\pi^{2}{\left(R\theta_{b}\right)}^{2}R^{2}}{T_{a}}^{2}T_{o}$$

and, by using $A_{rx}$ and F−number, the detected LiDAR power can be reduced to

(6) $$P_{d}\left(R,\theta_{b}\right)=\frac{\rho_{t}P_{s}{D_{rx}}^{2}A_{t}}{4\pi {\theta_{b}}^{2}R^{4}F^{2}}{T_{a}}^{2}T_{o}$$

where—since the receiver’s FOV is filled with the target—the detected power is affected by the whole target area $A_{t}$.

Maximum Range is limited fundamentally by the LiDAR signal-to-noise ratio (*SNR*). When the receiver FOV is filled, the *SNR* [21,22] is

(7) $$SNR=\frac{P_{d}}{NEP}=\frac{\rho_{t}P_{s}A_{d}{T_{a}}^{2}T_{o}}{4\pi R^{2}{\theta_{b}}^{2}{\left(F\right)}^{2}\left(NEP\right)}$$

where *NEP* is the Noise Equivalent Power, and the maximum range is

(8) $$R_{max}=\sqrt{\frac{\rho_{t}P_{s}A_{d}{T_{a}}^{2}T_{o}}{4\pi {\theta_{b}}^{2}{\left(F\right)}^{2}\left(NEP\right){SNR}_{th}}}$$

When the FOV is under-filled, *SNR* is

(9) $$SNR=\frac{P_{d}}{NEP}=\frac{\rho_{t}P_{s}{D_{rx}}^{2}A_{t}{T_{a}}^{2}T_{o}}{4\pi R^{4}{\theta_{b}}^{2}\left(NEP\right)}$$

where the minimum range is limited by how fast we can start sampling and by receiver–transceiver overlap

(10) $$R_{min}=\sqrt[{4}]{\frac{\rho_{t}P_{s}{D_{rx}}^{2}A_{t}{T_{a}}^{2}T_{o}}{4\pi {\theta_{b}}^{2}{\left(F\right)}^{2}\left(NEP\right){SNR}_{th}}}$$

### 2.1. Pulsed Time of Flight LiDAR

**Figure 3:**
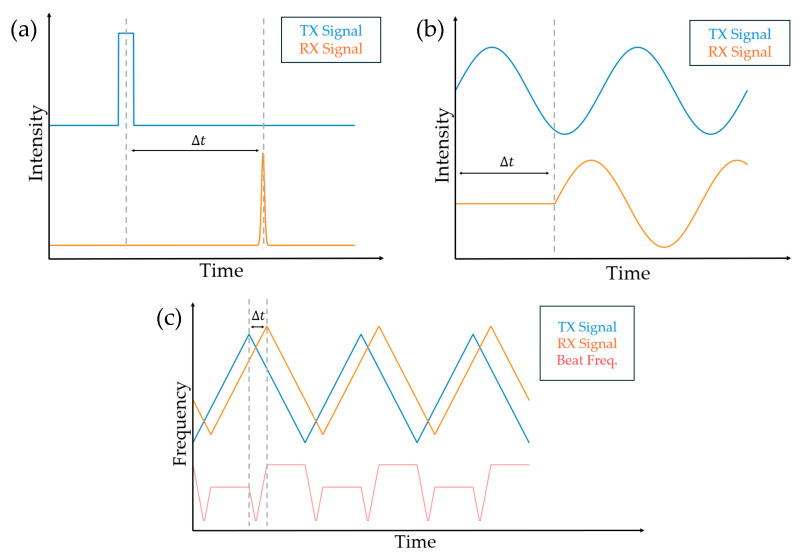
(**a**) In pulsed time of flight (TOF) LiDAR, pulse laser source reaches the object and reflects back to the detector. Time delay between source signal and detected signal determines the range of the object. (**b**) In amplitude-modulated continuous-wave (AMCW) TOF LiDAR, laser with continuous wave with oscillating amplitude or intensity is used. The phase difference between source and detected signal determines the range of the object. (**c**) In frequency-modulated continuous-wave (FMCW) LiDAR, frequency is chirped upward and downward in a triangular form. The time delay between the reference and received signal and the velocity of the object can be calculated from the measured beat frequency and the Doppler shift.

Time of flight works with the principle of measuring the time between the emitted optical pulse by TX (laser), reflected from the target object, and detected by the RX (detector). With this method, the distance R of the object can be calculated using the following equation:

(11) $$R=c\frac{\Delta t}{2}$$

where Δt is the time delay between the emitted light and detected light; and c is the speed of light (Figure 3a).

### 2.2. Amplitude-Modulated Continuous-Wave TOF LiDAR

Instead of a pulsed laser beam, amplitude-modulated continuous-wave (AMCW) [23] LiDAR utilizes a continuous laser beam. The laser beam’s amplitude or intensity is modulated by a sinusoidal wave or other waveforms. AMCW LiDAR measures the phase difference between the modulated amplitude of the transmitter and the one detected by the receiver.

(12) $$R=c\frac{\Delta t}{2}=c\frac{\Delta \varphi }{2\times \left(2\pi f\right)}=\frac{c\Delta \varphi }{4\pi f}$$

where Δφ is the phase shift; and f is the modulation frequency of the optical signal (Figure 3b).

### 2.3. Frequency-Modulated Continuous-Wave LiDAR

Frequency-modulated continuous-wave (FMCW) LiDAR, displayed in [24], uses a continuous laser beam whose frequency is oscillated over a specific range. FMCW LiDAR uses coherent detection, which is the comparison of the frequency with a reference signal from the transmitter. The interference of the reference signal and the light reflecting off a target generates a beat frequency, $f_{b}$, signal. The distance and the velocity of the moving object can be calculated from the beat frequency.

In the example of a triangular FMCW, frequency is chirped upward and downward within a range of bandwidth, B, over a modulation time, $T_{mod}$. The slope of the triangular curve γ is $2B/T_{mod}$. The measured upward beat frequency $f_{b,up}\;$ is decreased by the Doppler shift, $f_{shift}$ ($f_{b,up}=\;f_{b}-f_{shift}$), while the measured downward beat frequency is increased by the Doppler shift, ($f_{b,down}=\;f_{b}+f_{shift}$). The time delay between the reference and received signal is $\Delta t=\frac{T_{mod}f_{b}}{2B}$ with $f_{b}=(f_{b,up}+f_{b,down})/2$. Then the distance can be calculated as

(13) $$R=c\frac{\Delta t}{2}=\frac{c}{4B}\frac{f_{b,up}+f_{b,down}}{2}$$

The velocity of the target object can be calculated from the Doppler shift and the beat frequency relationship $f_{shift}={(f}_{b,down}-f_{b,up})/2$. $f_{shift}$ is ${\;2f}_{c}(v/c)$ by assuming the source velocity is zero.

(14) $$v=\frac{c}{2f_{c}}f_{shift}=\frac{c}{2f_{c}}\frac{{(f}_{b,down}-f_{b,up})}{2}$$

where $f_{c}$ is the starting frequency without chirping (Figure 3c).

## 3. LiDAR Scanning Platforms

### 3.1. Mechanical Rotating LiDAR

**Figure 4:**
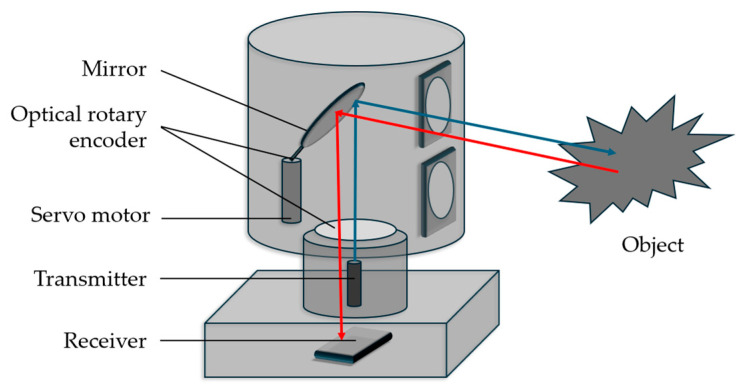
Diagram showing the function of a mechanical LiDAR system. Light from the laser source hits a 360° rotating mirror and scans the environment. Reflected signal from the objects is collected by the receiver’s optics, and the light is transferred to the receiver.

A mechanical scanning LiDAR system [25,26] is composed of a processor that controls a motor to spin a tilting mirror with a pulsed laser system to achieve a 360-degree field of view. The reflected beams from surrounding objects are then received by the tilting mirror and sent to a photodiode and amplified. In these devices, the laser diodes and photodiodes are stacked on top of each other, which limits the position of this device to the top of a vehicle. A diagram of a mechanical LiDAR device can be seen in Figure 4. The pros to this technology are that it is the oldest LiDAR system and has matured, although the bulky setup required limits its scalability. Without the right optical components, it can require multiple lasers and detectors, which can also make it very expensive.

### 3.2. Microelectromechanical LiDAR

**Figure 5:**
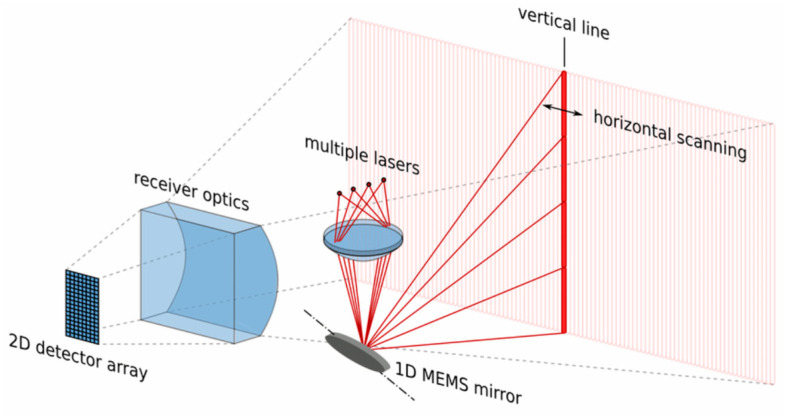
Diagram showing an example of a microelectromechanical LiDAR system. Lasers are collimated onto a 1D micromechanical (MEMS) mirror and directed through vertical scanning. Horizontal scanning can be achieved by positioning the MEMS mirror. Adapted with permission from [29] Copyright © IARIA.

Another type of LiDAR is known as micromechanical or microelectromechanical system (MEMS) [27,28] technology. This is a type of solid-state LiDAR where a MEMS-based mirror is used to scan the environment. An example of this can be seen in Figure 5, where a 1D MEMS mirror is used with multiple lasers to create a vertical line that can horizontally scan the environment. This technology is semi-mature but has improved scalability in fabrication compared to rotational LiDAR. It is also cheaper but more fragile.

### 3.3. Solid-State LiDAR

**Figure 6:**
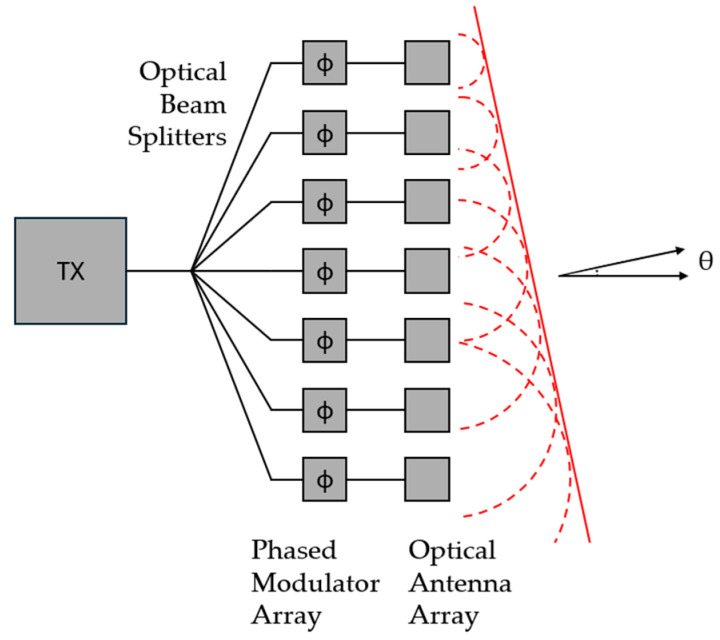
Diagram showing an example of an optical phased array (OPA) LiDAR system. Light from laser sources is transferred to phase modulator array through optical beam splitters. Phases are changed by applying voltage to each array. Modulated phase results in a deflection in the optical output beam.

Another type of solid-state LiDAR involves using integrated photonics with an optical phased array (OPA) [30,31]. An optical phased array consists of a laser and power splitters that split the light. The light then travels through an active phase modulator array, which modulates the phase of the light according to the applied voltage. The light can be sent in a different direction simply by electrifying the array, and does not require mechanical movement. A diagram of an optical phased array can be seen in Figure 6. OPA systems are the cheapest and most robust LiDAR systems. The fabrication technology to create these on silicon is very mature, which also allows for high scalability of fabrication, although the design technology for these devices is limited and semi-mature.

## 4. Metasurfaces

Metasurfaces are engineered into two-dimensional structures that are composed of periodic or quasi-periodic arrangements of subwavelength elements that can manipulate the amplitude, the phase, or the polarization of light at their interface. Unlike traditional three-dimensional metamaterials, which are challenging to fabricate, metasurfaces offer a planar platform and compact form. Therefore, they are easier to integrate into other optoelectronic and photonic systems [32,33,34,35]. Based on the principles of diffraction and interference, these metasurfaces can be tailored to achieve functionalities such as anomalous reflection and refraction, beam shaping, polarization conversion, and wavefront control with high precision by locally varying the geometry, orientation, or material properties of each element. These functionalities of metasurfaces have enabled improvements in applications across a wide range of fields, including telecommunications, quantum photonics, biomedical devices, imaging, and sensing [34,36,37,38].

Metasurfaces have been widely fabricated in research labs by using electron beam lithography, which is precise but slow and expensive, limiting scalability. Recent studies show that CMOS-compatible fabrication using deep-ultraviolet (DUV) lithography, reactive ion etching (RIE), and atomic layer deposition (ALD) enables large-area, high-volume, and cost-effective metasurface production [39,40,41,42,43,44].

### 4.1. Phase Shift, Deflection Angle, and Field of View

When objects are at the wavelength or subwavelength scale, such as in metasurfaces, a generalized Snell’s law is used to account for the change in phase across the object. This generalized equation includes a phase-gradient (shift) element [45]:

(15) $$n_{t}\sin\left(\theta_{t}\right)-n_{i}\sin\left(\theta_{i}\right)=\frac{\lambda_{0}}{2\pi }\frac{d\Phi }{dx}$$

where $\theta_{i}$ is the incident angle (in this case zero); $\theta_{t}$ is the transmitted angle; $n_{t}$ and $n_{i}$ are the refractive indices of the material; $\lambda_{0}$ is the wavelength of light; and $\frac{d\Phi }{dx}$ is the phase gradient—the total change in phase (F) over a unit cell (x). When light interacts with the boundaries of an object with dimensions all larger than $\lambda_{0}$, the phase function Φ(*x*) is constant and the right-hand side of the equation is zero. We regain the traditional form of Snell’s law, $n_{t}\sin\left(\theta_{t}\right)=n_{i}\sin\left(\theta_{i}\right)$, and the light experiences ordinary refraction.

Baker et al. calculated the phase shift of the metasurface array from the real and imaginary parts of complex transmission S_21_ by using the relationship [46]:

(16) $$\Delta \Phi =atan\left[\left(\frac{Im(S_{21})}{Re(S_{21})}\right)\times \left(\frac{1}{\pi }\right)\right]$$

For a complete array, manipulating the generalized Snell’s law yields a propagation angle for outgoing light.

(17) $$\theta =\sin^{-1}\left(\frac{\lambda }{\Lambda }\right)$$

This is based on the grating equation:

(18) $$m\lambda =\Lambda \left(\sin\theta_{t}-\sin\theta_{i}\right)$$

where m is the mode of light; and Λ is the length of the total array. However, this assumes a linear phase function varying from 0 to 2π across the system. The phase can be calculated from the real and imaginary parts of the complex transmission using the relation $\Delta \Phi =atan[\left(\frac{Im(S_{21})}{Re(S_{21})}\right)\times \left(\frac{1}{\pi }\right)]$. The phase change ΔΦ is incorporated into the final θ equation:

(19) $$\theta =\sin^{-1}\left(\frac{\lambda }{\Lambda }\times \frac{\Delta \Phi }{2\pi }\right)$$

where the variables include the total phase shift ΔΦ and the total array length (Λ).

**Figure 7:**
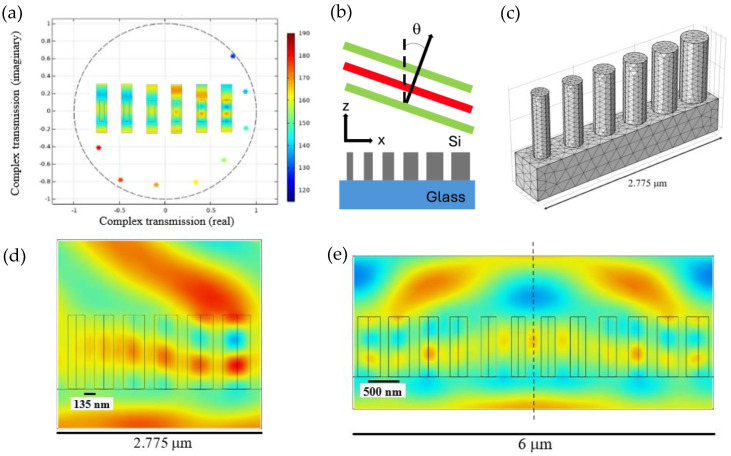
Silicon metasurface design for beam deflection. (**a**) Simulated complex transmission for cylindrical silicon post for various radii as the color bar on the side shows. Inset shows the electric field profile for each radius. (**b**) Schematic design of the beam deflector metasurfaces. (**c**) Showing the physics-controlled mesh used in silicon post array. (**d**) Deflected electric field profile for six silicon posts with increasing radius. E-field confined more towards the outer rods with higher radius shows the beam deflection towards right. (**e**) The six-post array is mirrored around the mid-axis to increase the field of view (FOV). (**a**–**e**) Adapted with permission from [46]. Copyright © 2025 Optica Publishing Group.

Baker et al. used COMSOL Multiphysics 5.6b software, wave optics module, frequency domain, to design and simulate the beam-deflecting metasurfaces. First, in a 500 nm unit cell, a 1-micrometer-thick single cylindrical silicon (n_Si_ = 3.48) post with a radius of 115 nm is built on a 537-nanometer-thick silicon dioxide (n_SiO2_ = 1.44) substrate. Port 1 at the bottom served for incoming light with E_y_ = 1 V/m and P_in_ = 1 W, Port 2 at the top was designated for outgoing light, and at the other four sides, Floquet periodicity was used. Complex transmission is plotted for different radii ranging from 115 nm to 190 nm in Figure 7a, and the inset shows the E-field profile for each radius value. Next, six silicon posts with varying radius from 115 nm to 200 nm with a step size of 15 nm were built on a silicon dioxide wafer. The edge-to-edge spacing between each rod is 135 nm, and the E-profile is shown in Figure 7b.

The light wavelength is confined in the outer rods and loosely confined in the rods with smaller radii. This effect results in a beam deflection at Port 2 with 79.6% transmission. The phase shift is estimated as 1.35π, and the deflection angle is calculated as 22.2°. The downside of edge-to-edge spacing is that the period between each post is changing; this would be considered as non-uniform (chirped) grating, and the grating equation no longer applies and can only be used as an estimate. Another design consists of six posts with radii ranging from 90 nm to 195 nm and a spacing of 500 nm between the centers of the silicon rods. This design yields a greater deflection angle of 24.8° compared to the previous design due to having a larger phase shift of 1.62π. Finally, removing the second post with 130 nm decreased the distance without altering the phase change. The deflection angle is calculated as 30.17°. The final design shows the effect of increasing the angle without sacrificing much confinement as the transmission reduces from 82% to 78.6%. To have a wider field of view (FOV), the design with five silicon posts was mirrored around the middle of the axis. Figure 7c shows that the electric field component is deflected towards both top-right and top-left corners, yielding a wider scan area with a total transmittance of 83.2%. This is a promising design for increasing the FOV in solid-state LiDAR for autonomous vehicles.

### 4.2. Passive Metasurfaces

Metasurfaces are fabricated through advanced lithography techniques such as electron beam lithography, plasma etching, and/or atomic layer deposition. After fabrication, depending on the design criteria, some of the metasurfaces will have a fixed optical electromagnetic response. These are called passive metasurfaces. On the other hand, if the metasurface is designed to alter the functionality by external stimuli after the fabrication, these structures are called the tunable metasurface.

Passive metasurfaces provide static, high-performance wave control for specific applications like lenses, filters, and polarizers with pre-defined functions. Passive metasurfaces are still used in beam-deflecting applications as metalenses, offering a wide FOV over 150° [47,48]. In addition to achieving a wide FOV, passive metasurfaces are used for echo-signal band-pass filtering [49,50,51,52], beam splitting and combining [47,53,54,55,56,57], and beam shaping [52,58,59,60,61,62].

**Figure 8:**
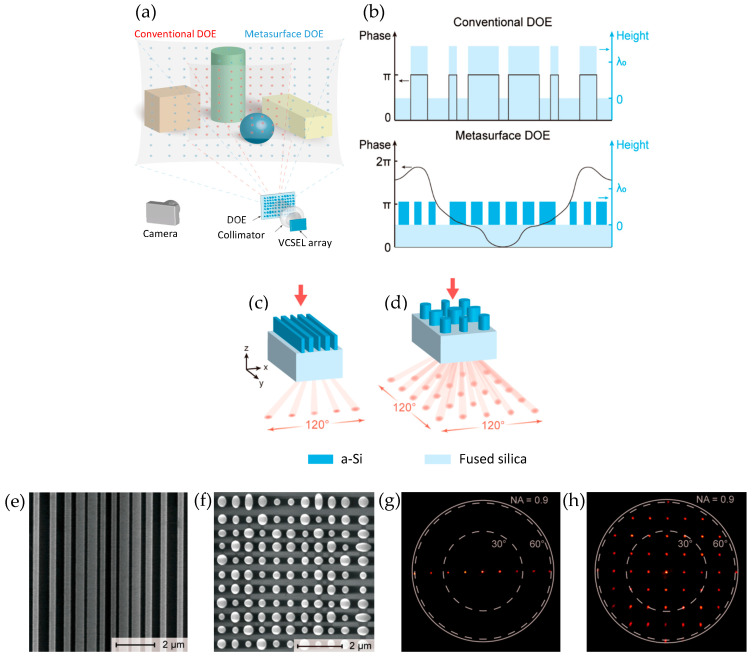
(**a**) Working principle of conventional and metasurface diffractive optical element (DOE) projection. (**b**) Phase profile comparison of conventional DOE with binary phase profile and metasurface DOE with adjustable phase profile. Designed metasurfaces for (**c**) 1D with bars (**d**) and 2D with cylindroid posts. Scanning electron microscope (SEM) top-view images of these (**e**) 1D and (**f**) 2D structures. (**g**) One-dimensional and (**h**) two-dimensional spot arrays produced by these metasurfaces. (**a**–**h**) Adapted with permission from [48]. Copyright © 2020 American Chemical Society.

Wide FOVs can be achieved by using the metasurface as a wide-angle beam projector. Lin et al. [49] achieved a metasurface-assisted wide-angle beam projector with a 120° FOV for operation at 1550 nm wavelength. Cylindrical meta-atoms of various radii and height, made of amorphous Si deposited on a glass wafer substrate, were optimized to provide an optical phase delay from 0 to 2π while maintaining 90% or greater transmission. Metasurfaces were fabricated using plasma-enhanced chemical vapor deposition (PECVD) of amorphous Si on glass wafers, electron beam lithography, plasma etching, and an epoxy coating to cover the surface, producing a defect-free metasurface. This metasurface was bonded to an optical aperture and diced, then coupled to a PIC. A setup of an optical rail with an IR camera and CMOS image sensor mounted on a goniometer was constructed and aligned to the optical axis of the collimated beam. The beam-steering angle was measured by the goniometer, and the beams’ cross-section was captured to characterize beam size and divergence angle. Another way of achieving a wide FOV is using diffractive optical elements (DOEs). Typically, DOEs have low efficiency and low uniformity for large FOVs [63]. Researchers like Ni et al. [48] demonstrate a silicon-metasurface-based diffractive optical element (DOE) for structured light projection for an over 120° by 120° field of view, designed for 1550 nm. The authors prove that polarization-independent silicon-based metasurfaces can be used to produce a far-field spot array in large FOVs from a collimated laser beam. Using methods such as vectorial electromagnetic simulation and interior point method for optimization, the size and position of meta-atom posts can be adjusted to design structured light projectors with an efficiency of 96% and 81% and a root mean square error (RMSE) of 5% and 24% for 1D and 2D, respectively, on par with modern DOEs with lower FOVs. The polarization-dependence of 2D spot projection can be eliminated by utilizing supercell metasurfaces with four-fold rotational symmetry. Metasurfaces were fabricated using electron beam lithography and reactive ion etching. These devices were tested using a Fourier space imaging setup and achieved experimental efficiencies of 56% and 59% and RMSEs of 40% and 39% for 1D and 2D, respectively. The cause of these lower efficiencies and higher RMSEs could be a low tolerance to fabrication, which could be improved with further optimization and adjustments for fabrication tolerance (Figure 8). 

In LiDAR, for precise distance measurement and object detection, it is crucial to isolate the return signal (echo) at a specific wavelength to filter out the background light and unwanted spectral components. Echo-band-pass filtering metasurfaces enable the selection of specific wavelength bands and improve the signal-to-noise ratio and accuracy [64]. By allowing only the desired echo band to pass, these metasurface filters suppress the background noise and spectral crosstalk [65]. Ultra-narrow bandpass filters with out-of-band suppression capability are significant in the field of near-infrared spectroscopy. Grating-based guided-mode resonance filters (GMRFs) have received much attention in research for their high diffraction efficiency and narrow-bandwidth characteristics. GMRFs operate by coupling input light through a grating waveguide layer into the leaky Bloch mode, exciting the GMR perpendicular to the waveguide’s grating slits to achieve ultra-narrow bandpass filtering. There are multiple ways to achieve this, and Hu et al. [66] demonstrate a design method for single-layer GMR narrowband bandpass filters that can achieve single-channel filtering for a bandwidth up to 400 nm, with a range from 1350 to 1750 nm. This is achieved through the incident TM mode light that is coupled into the leaky Bloch modes through periodic groove perturbations, and the resulting interference between the groove radiation produces the narrowband resonance. The design method for this grating begins with the Particle Swarm Optimization (PSO) algorithm to optimize period, thickness, and filling factor. Performance can be optimized using the root mean square fitness function, guaranteeing numerical quality. The linear spectrum and interaction between electromagnetic waves and periodic grating structures of the designed structure are then calculated using the RCWA method. Structures are created in three stages: first, the base grating (TMG) is established to provide broadband transmission suppression, then periodic groove perturbations are introduced to couple the incident light into high-Q guided modes propagating parallel to the grating slits, before redundant resonances are eliminated with positive and negative interference, leaving a single high-Q resonance. Simulated structures consist of silicon nanogratings on a SiO_2_ substrate and assume infinite periodic structures, lossless materials, and perfectly matched layers. Simulated designs achieve a Q-factor of over 100,000, attenuation below 1% outside the passband, and are highly robust against fabrication defects as the resonance peak position shifts less than 0.5 nm.

**Figure 9:**
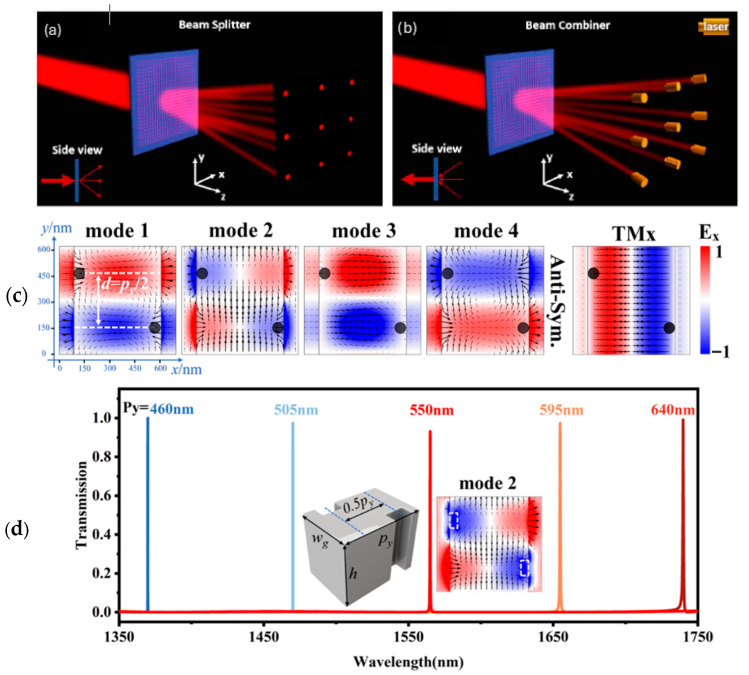
(**a**) Beam splitter metasurface furcating a collimated beam into nine beam paths arranged into a 3 × 3 grid. (**b**) The inverse process in which nine laser beam sources are arranged along the furcated beam paths towards the metasurface to combine the nine laser beams into one larger collimated beam. (**a**,**b**) Adapted with permission from [68] Copyright © 2023 Optica Publishing Group under the terms of the Optica Open Access Publishing Agreement. (**c**) Mode electric field simulations in the xy plane for sample structure. (**d**) Transmission spectra of double-groove sample structure with different groove periods, listed above. (**c**,**d**) Adapted with permission from [66]. Copyright © 2024 Optica Publishing Group under the terms of the Optica Open Access Publishing Agreement.

Passive metasurfaces are ultra-thin, planar optical elements that can split or combine light beams with high precision. In LiDAR systems, these functionalities are crucial for directing, multiplexing, or demultiplexing laser signals, which are essential for 3D imaging, sensing, and communication [67]. Conventional metasurface-based splitters often suffer from low efficiency and poor uniformity across output beams. Recent designs using advanced optimizations such as inverse designs achieve above 90% efficiency and above 97% uniformity over broad bandwidths [55,56,68]. Hemayat et al. [68] demonstrate the design and simulation of a highly uniform, broadband metasurface beam splitter and combiner based on dielectric nanostructures. Metasurfaces, ultra-thin optical elements that manipulate light through subwavelength “meta-atoms”, offer miniaturization advantages over conventional optics; however, most metasurface beam splitters suffer from narrow bandwidths and non-uniform diffraction orders, limiting their practical performance. To address these issues, the authors developed a reciprocal metasurface capable of both beam splitting and combining with incredible efficiency and uniformity across a 50 nm wavelength range (1525–1575 nm) (Figure 9a,b). The device achieves >97% uniformity and >90% diffraction efficiency (≈88% in combiner mode) at the 1550 nm telecom wavelength. The design is inspired by Dammann gratings, known for generating uniform diffraction patterns, but realized here through a Pancharatnam–Berry-phase metasurface made of amorphous silicon elliptical pillars on a $SiO_{2}$ substrate. Each meta-atom’s rotation controls the local optical phase without altering geometry, simplifying fabrication. To overcome phase discretization errors that degrade uniformity, the team employed a modified Particle Swarm Optimization (PSO) algorithm. Their algorithm integrates Latin hypercube noise injection and mirrored local search to escape local minima, optimizing 729 meta-atom rotation angles. This method improved the 2D beam splitter’s diffraction efficiency by 16.5% and uniformity by 15.5%, outperforming traditional metasurface and Dammann designs. The results demonstrate a numerically optimized, fabrication-friendly approach for compact, broadband optical beam management. Such metasurfaces hold promise for high-power laser systems, quantum photonics, LiDAR, and optical sensing, where near-perfect uniformity and high efficiency are essential.

Passive metasurfaces are used for focusing, collimation, deflection, and generating complex beam profiles. They can generate custom beam profiles such as collimated, Bessel, vortex beams, and direct beams at specific angles that are difficult or impossible with conventional optics [41,56,60]. They can achieve wide-angle beam deflection [41]. Choi et al. [58] demonstrate a computationally optimized metasurface platform capable of generating 360° structured light for compact, solid-state depth imaging. Using a differentiable optical model that can accurately simulate 180° far-field propagation without paraxial approximations, the team optimizes the metasurface phase profile with task-oriented neural networks, achieving holographic propagation speeds over 50,000× faster than traditional numerical propagation methods. The device employs hydrogenated amorphous silicon metal atoms on a silica substrate to realize identical structured-light patterns in both reflection and transmission, enabling omnidirectional 3D illumination without mechanical motion. Experimental results show active-stereo depth imaging with a mean error of 3.5 cm across a 2.5 m range. Offering more than five-fold accuracy improvement over conventional, diffractive, or multi-dot projectors. For LiDAR applications, this approach provides a path toward fully solid-state, wide-field beam-steering and depth-sensing by merging learned optical design with nanophotonic hardware. The integration of the metasurface phase control and machine learning-based optimization directly addresses LiDAR’s key limitations in field of view, system size, and scanning speed while maintaining scalability for near-infrared and polarization-selective operations. Overall, the work establishes a critical foundation for computational metasurface architectures in next-generation LiDAR, robotics, and autonomous vision systems.

**Figure 10:**
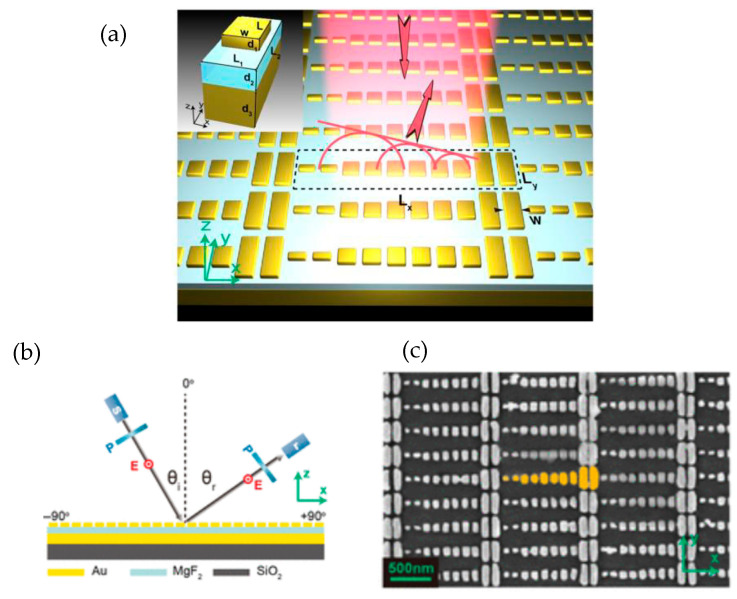
(**a**) Bird’s eye view of designed metasurface with a cross-section of a unit cell consisting of Au nanorod, MgF_2_ spacer, and continuous Au film from top to bottom. (**b**) Experimental setup, where “s,” “r,” and “p” represent source, receiver, and polarizer. (**c**) SEM overhead image of fabricated metasurface. One supercell is highlighted in yellow. (**a**–**c**) Adapted with permission from [69]. Copyright © 2012 American Chemical Society.

Metamaterials (MTMs) are artificial materials that manipulate the propagation of light with their parameters of permittivity and permeability. Conventional homogeneous and slow-varying inhomogeneous metamaterials usually use adiabatic special changes in permittivity and permeability to manipulate light inside the medium while being much larger than the wavelength. Novel abruptly varying metasurfaces are ultra-thin, which can fix the issue of scattering loss and phase distortion caused by the size of the metamaterials (MTMs). Sun et al. [69] used metasurfaces of V-shaped antennas for producing anomalous reflections and refractions using the generalized Snell’s law with 100% efficiency of converting propagating waves to surface waves. These novel metasurfaces also provide greater control over the propagation and wavefronts of light. However, some issues persist in the IR range. These V-shaped antenna metasurfaces support normally reflected and refracted beams along with the desired anomalous ones, lowering the efficiency of the light manipulation. Anomalous beams also possess different polarizations than the input light. Sun et al. demonstrate a new metasurface design to redirect light into a single anomalous reflection beam, with polarization and conversion efficiencies being as high as 80% to overcome these issues for light around 850 nm, and an operation bandwidth exceeding 150 nm. The device consists of a Au plate with 130 nm height under a 50-nanometer-thick MgF_2_ spacer where antennas sit, making it much smaller than the operational wavelength. This extra layer blocks transmitted light, forcing the metasurface to act in reflection mode. Reflected amplitude has low variance between units, so the only issue left is the phase delay of reflected light, which can be tuned by adjusting the antenna length. Antennas on the surface vary from 40 nm to 260 nm in length, optimized to simulated reflected field patterns. In the experiment, fabricated devices proved to operate similarly and as successfully as simulations, with about 80% of light converted to the anomalous mode with one intensity peak and the rest being absorbed—a large improvement over past devices (Figure 10). The details of passive metasurfaces reviewed in this section can be viewed in Table 1. 

### 4.3. Tunable Metasurfaces

Tunable metasurfaces provide dynamic, non-mechanical beam steering and wavefront control for compact, high-speed, and high-resolution LiDAR systems. The optical properties of metasurface materials can be dynamically controlled by external stimuli such as electrical gating, thermal variation, optical stimulation, and mechanical actuation to achieve rapid, efficient, and precise modulation of light [50,51,52,53].

**Figure 11:**
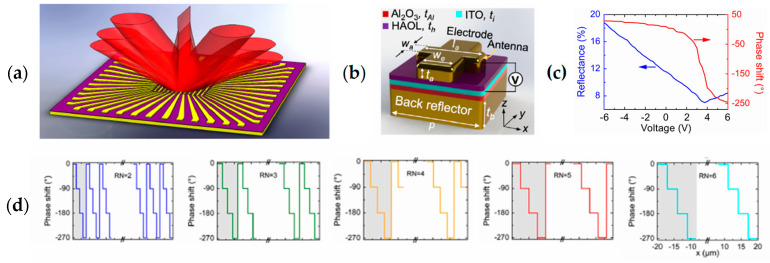
(**a**) Tunable changes in beam direction of up to 270° without mechanical motion due to the metasurface’s field-effect modulation. (**b**) The metasurface design of layered materials, including a Au back reflector, $Al_{2}O_{3}$ dielectric, ITO active layer, and Au “fishbone” antennas separated by a high-permittivity hafnium/aluminum oxide nanolaminate (HAOL). (**c**) Measured reflectance (blue curve) and phase shift (red curve) as a function of applied bias voltage. (**d**) Spatial phase distributions of the metasurface elements with different repeat number (RN) values that are used to create phase gradients resulting in beam steering. (**a**–**d**) Adapted with permission from [70]. Copyright © 2020 American Chemical Society.

Electrical gating is a widely used method where an external electrical voltage or current is applied to modify the carrier concentration and molecular orientation. Some of the mechanisms used for electrical tuning are field-effect modulation [70,71,72,73], electro-optic effect [50,58,74], phase-change materials [51,75,76], and liquid crystal (LC) reorientation [77]. Applying voltage across the active materials, such as indium tin oxide (ITO), graphene, and other conductive oxides, changes the carrier concentration at the interface, altering the refractive index and enabling the phase and amplitude modulation. Shirmanesh et al. [70] introduce a multifunctional metasurface composed of 96 independently addressable nanoantennas, each capable of modulating the phase of reflected light through applied electrical bias. The core mechanism relies on field-effect modulation of indium tin oxide (ITO) in the epsilon-near-zero (ENZ) regime, which enables large, reversible phase shifts exceeding 270° without mechanical motion (Figure 11a). The metasurface design of layered materials includes a Au back reflector, $Al_{2}O_{3}$ dielectric, ITO active layer, and Au “fishbone” antennas separated by a high-permittivity hafnium/aluminum oxide nanolaminate (HAOL) (Figure 11b). Applying a DC voltage alters the carrier density in ITO, dynamically changing its refractive index and, consequently, the phase of reflected light (Figure 11c). The device is fabricated using electron-beam lithography and atomic layer deposition, integrating it with custom-printed circuit boards to individually control each element via computer-programmed microcontrollers. Two primary optical demonstrations highlight the metasurface’s capabilities. First, beam steering was achieved by imposing a programmable spatial phase gradient across the antenna array (Figure 11d). Adjusting the voltage patterns allowed the reflected beam to deflect up to approximately 22° experimentally, consistent with simulations predicting up to 70° depending on the array pitch. Second, by reprogramming voltage distributions, the same device acted as a dynamic focusing meta-mirror, capable of switching focal lengths between 1.5–3 µm and 15–25 µm experimentally. The first programmable multifunctional metasurface that can be dynamically reconfigured between distinct optical operations via electronic control was demonstrated, and the technology points toward compact, high-speed, energy-efficient optical systems such as on-chip LiDAR, holography, and adaptive imaging.

Another important work on phase change was conducted by Forouzmand et al. [71]. A reflectarray is an array of reflecting elements capable of adjusting the reflection phase from each element to form the desired wavefront. Electrically tunable metasurfaces are vital for reflectarray devices as they allow for control over the beam profile. Conductive and transparent materials like ITO allow for adjustment of the phase, for example in metal–insulator–metal (MIM) structures. Carrier accumulation at the ITO–Al_2_O_3_ interface changes the plasma frequency and, therefore, the permittivity and refractive index, leading to phase tunability. Some methods of integrating these materials—like ITO, for example—into a metasurface include an ITO accumulation layer in an MOS multilayer stack on a Si waveguide. Alternatively, an ITO-insulator layer can be embedded with metal from both sides to form MIM structures. Light can be confined in these structures and achieve absorption or manipulate light due to their Fabry–Perot-like resonance. A thin layer of ITO on an array of gold nanoantennas can achieve tunable infrared absorption. Forouzmand and Mosallaei demonstrate an electrically tunable reflectarray device with ITO-integrated MIM plasmonic patch antennas enabling 2D beam steering over parallel and perpendicular polarizations in the NIR. Unit cells of patch nanoantennas on the ITO layer scale to form the plasmonic reflectarray metasurface. Voltage adjustment of each unit creates a phased array to control the beam-steering angle. Wires connect to each unit cell, and DC voltage is applied from one side of the device. This device consists of square-shaped 50 nm gold nanoantennas with a width of 320 nm and period of 500 nm on a 12 nm Al_2_O_3_ layer, a 26 nm ITO layer, and a 100 nm gold layer from top to bottom. Voltage is applied from the top nanoantenna to the gold back mirror. Simulations revealed that the 20 by 20 nanoantenna array showed side lobes, a wide profile of the main lobe, and a shift in intensity peak as the beam-steering angle increased. These destructive effects are not significant enough to have a large effect but can be diminished by increasing the size of the array. Another important work on a gate-tunable conducting oxide metasurface capable of dynamic electrical control over both the phase and amplitude of reflected near-infrared light is demonstrated by Huang et al. [72]. Their design utilized a metal oxide semiconductor (MOS) configuration comprising a gold backplane, a thin Al_2_O_3_ dielectric layer, and a thin indium tin oxide (ITO) active layer patterned with gold stripe antennas. By modulating the gate voltage between the antenna and backplane, the carrier concentration at the Al_2_O_3_/ITO interface could be tuned to induce charge accumulation or depletion, thereby altering the complex refractive index of the ITO. When biased near its epsilon-near-zero (ENZ) regime—where the real permittivity approaches zero—strong coupling between the antenna’s magnetic dipole resonance and the ENZ region enabled efficient control of reflected light properties. Experimentally, the metasurface achieved a phase modulation of approximately 184° and a reflective variation of about 30% under a gate bias of 2.5 V, with measured modulation frequencies up to 10 MHz and theoretical limits extending to gigahertz or terahertz operation due to the low capacitance of the structure. Furthermore, the authors demonstrate an electrically tunable phase grating by selectively gating subsets of antennas, allowing for beam steering over angular ranges exceeding 40° without any mechanical components. This work represents one of the earliest experimental validations of electrically reconfigurable metasurfaces, offering a compact, low-power, and high-speed alternative to conventional beam-steering mechanisms. The demonstrated approach is highly relevant to solid-state LiDAR architectures, where precise, rapid, and non-mechanical control of optical wavefronts is essential. Conducting-oxide-based metasurfaces such as this hold significant promise for next-generation integrated optical systems, including dynamic holography, adaptive focusing, and nanoscale spatial light modulators for LiDAR sensing and imaging applications [72].

Bikbaev et al. [78] proposed a device integrating a metasurface of gold nanobricks atop a one-dimensional photonic crystal (PhC) separated by thin layers of sapphire, graphene, and ITO. The graphene monolayer serves as a transparent electrode, while the ITO layer’s carrier density changes under electrical bias, causing its dielectric permittivity to shift from positive to negative in the epsilon-near-zero regime. This transformation induces significant phase variation in the reflected wave of over 200° at telecom wavelengths (~1550 nm), allowing for active optical control. Finite-difference time-domain (FDTD) simulations confirmed that the structure’s critical coupling condition is reached at about 4 V, producing strong TPP resonance with a four-fold increase in quality factor compared to conventional metal–insulator–metal systems. Increasing the bias voltage blue-shifts the TPP resonance and modulates the reflection phase, enabling beam deflection in both polar (ϕ) and azimuthal ($N^{2}$) directions. The metasurface acts as a dynamic phase diffraction grating, generating reconfigurable diffraction orders through programmable phase profiles applied to each nanobrick. Two control architectures were analyzed, one with independent contacts for full phase control of each element, and another with 2N contacts for simplified implementation. While the 2N scheme slightly reduces diffraction intensity (~15% lower), it maintains high performance for small deflection angles (<6°). This balance highlights the trade-offs between beam control precision and system complexity. The study demonstrates a practical approach for electrically tunable, non-mechanical beam steering based on TPPs. The system achieves real-time modulation of light directionality in two dimensions, presenting strong potential for solid-state LiDAR dynamic holography and on-chip communication technologies. In another work, Bikbaev et al. [79] integrate a geometric-phase metasurface made of antimony trisulfide ($Sb_{2}S_{3}$) nanobricks with a metal–photonic crystal (PhC) system supporting TPPs. TPPs are localized optical states that form at the interface between a metal and photonic crystal, allowing for sharp resonant behavior and strong field containment. The metasurface exploits the Panchratnam–Berry phase, which arises from spatially varying orientations of anisotropic nanobricks. Each meta-atom rotation modifies the local optical axis, introducing a geometric phase shift proportional to twice the rotation angle, independent of the optical path length. $Sb_{2}S_{3}$ is used as a phase-change material, switching between amorphous and crystalline states with distinct refractive indices (from 2.77 to 3.49 at 1550 nm). Finite-difference time-domain (FDTD) simulations reveal that this transition enables strong modulation of reflected phase and intensity. When coupled with gold and a PhC tuned to telecom wavelengths, the structure achieves critical coupling at 1550 nm, producing a sharp TPP resonance with exponentially decaying fields on both sides of the interface. The key finding is that the diffraction order intensity depends on both the polarization and phase state of $Sb_{2}S_{3}$. In the amorphous phase, most energy resides in the zeroth diffraction order. Upon crystallization, left- and right-circularly polarized light produce opposite intensity redistributions between +1 and −1 diffraction orders. This polarization-dependent diffraction demonstrates controllable chiral selectivity and dynamic beam manipulation.

In addition to transparent oxide materials, graphene plasmonic metamolecules can be used to achieve complete complex amplitude modulation. Han et al. [73] present a major advancement in active metasurface design, enabling independent and continuous control of both the amplitude and phase of light. Traditional dynamic metasurfaces generally modulate only the phase of an optical wave, limiting the ability to fully shape wavefronts for applications such as holography, adaptive imaging, and more notably, beam steering. The inability to tune amplitude and phase separately often results in optical distortions and reduced efficiency. To overcome these limitations, the paper demonstrated designs of a two-parameter metamolecule composed of two graphene plasmonic ribbons (GPRs) coupled to metallic nanoantennas, where each GPR’s Fermi level can be independently adjusted via electrostatic grating. This dual-gating mechanism allows for simultaneous and decoupled tuning of phase and amplitude responses. Operating at a wavelength of approximately 7 µm, the device demonstrates both a full 2π phase shift and a broad range of amplitude modulation, including perfect absorption. The authors of the paper Han et al. [73] introduce a graphical admittance model that maps the relationship between surface impedance, Fermi levels, and optical response, providing a clear visualization of the conditions necessary for full complex modulation without requiring extensive numerical simulations. Experimental demonstrations confirm the design’s versatility: the metasurface achieves dynamic beam steering at controlled reflection angles (20.9°, 45.5°, and 72.0°) with consistent amplitude, and reconfigurable holographic focusing at single and multiple focal points with subwavelength precision. The study further explores the influence of graphene carrier mobility, showing that even at moderate mobility values around (500 cm^2^ V^−1^ s^−1^), effective amplitude and phase control remain attainable through optimized geometry.

**Figure 12:**
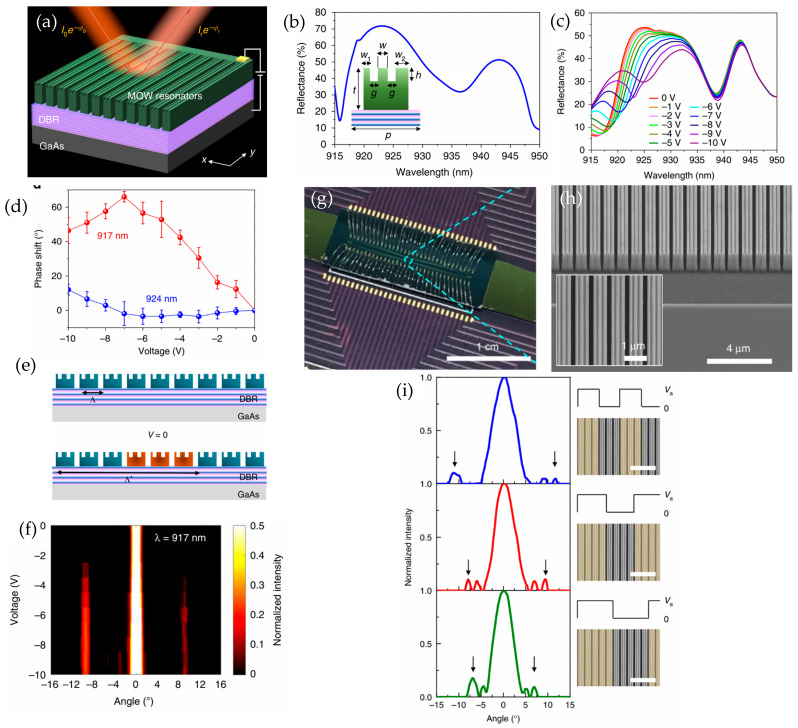
(**a**) Schematic diagram of the beam-steering device using all-dielectric III-V multi-quantum-well (MQW) metasurfaces. (**b**) Resonance wavelength of the structure under no applied bias computationally. Inset shows the double-slit structures are etched into the MQW layers. (**c**) Resonance wavelength response under applied bias from 0 V to −10 V experimentally. (**d**) The measured phase shift under the applied bias. (**e**) Applied voltage at every other three MQWs altered the period of metasurfaces. (**f**) The first-order diffraction pattern for applied voltage. (**g**) Photographic image of the dynamic beam-steering device based on gate-tunable metasurfaces. (**h**) Scanning electron microscopy image of the metasurfaces with electrical insulation. (**i**) Measured beam steering by dynamically changing the periodicity of the metasurfaces. (**a**–**i**) Adapted with permission from [50] Copyright © 2019 The Author(s).

An electro-optic effect is observed when an electric field is applied to materials like lithium niobate and III-V materials. The refractive indices of materials change due to the Pockels effect or quantum-confined Stark effect [50,59,74]. Wu et al. [50] demonstrated beam steering by applying an electrical bias to all dielectric III-V multiple-quantum-well (MQW) metasurfaces. The device structure consists of a GaAs substrate, an AlGaAs/GaAs distributed Bragg reflector (DBR), and an MQW layer, as shown in Figure 12a. Double-slit structures are etched into the MQW layer, as shown in the inset of Figure 12b. Figure 12b shows the resonance wavelength of the structure, under no applied bias, at around 915.9 nm and 936.3 nm computationally. Figure 12c shows the resonance response under the applied bias from 0 V to −10 V experimentally. When the applied voltage decreased from 0 V to −10 V, a significant red shift of the shorter-wavelength resonance was observed. Also, at 917 nm, an increase in the reflectance intensity was observed with decreased bias. The phase shift of the reflected beam was measured under the applied bias at a wavelength of 917 nm and 924 nm using a Michelson interferometer system, as shown in Figure 12d. The phase shift increases continuously by 70% from 0 V to −7 V bias. The phase modulation is weaker for the wavelength of 924 nm as the applied voltage decreases from 0 V to −10 V. This is especially true for the wavelengths away from the Mie-guided-mode resonance wavelength. Electrical switching of beam diffraction was achieved by applying bias to the metasurfaces by grouping them into elements of three (Figure 12e). This increased the effective periodicity of the metasurfaces from the subwavelength range (p = Λ = 910 nm) to a grating range ($\Lambda^{\prime }$ = 6 × p = 5460 nm). For the resonance wavelength of 917 nm, the first-order diffraction pattern appears for applied voltages below −3 V at an angle of about 9.66° (Figure 12f). In addition to the switchable beam diffraction, beam steering was demonstrated experimentally by controlling metasurface elements individually. By fully etching the air between the quantum well slabs, each unit element is electrically isolated (Figure 12g,h). The reflected beam was steered by applying an identical electrical bias to each metasurface element, which consists of three MQWs. The first-order diffraction angle becomes smaller as the periodicity of metasurface is increased, as shown in Figure 12i. This was achieved as the number of MQWs increases in each metasurface element that is under electrical bias. Achieving dynamic beam steering by electrically controlling each unit element over the MQW metasurface array provides a fundamental concept for future on-chip LiDAR systems.

**Figure 13:**
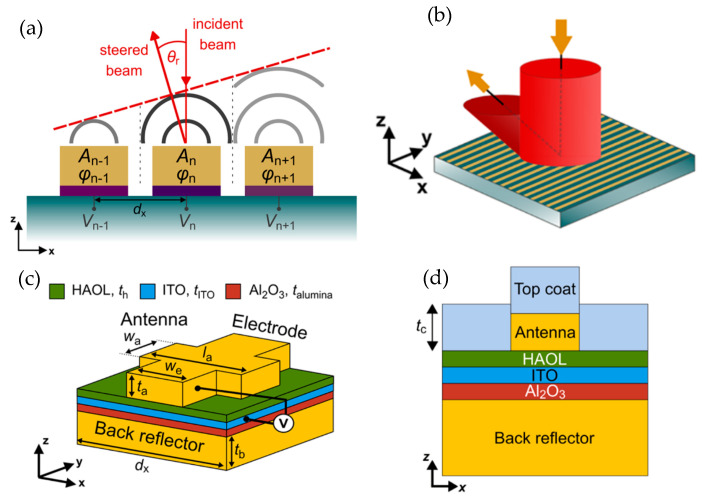
(**a**) Cross-section of active metasurface where applied voltage effects beam amplitude and phase, allowing for beam steering. (**b**) Bird’s eye view of 1D tunable metasurface. Antennas connected in y direction allowing for control in the x direction. (**c**) Example cross-section of unit cell of metasurface. (**d**) Schematic fabricated metasurface with SiO_2_ topcoat layer. (**a**–**d**) Adapted with permission from [80]. Copyright © 2020 American Chemical Society.

Inverse design is an advanced method for optimizing characteristics of photonic structures. For metasurface design, inverse design is usually used to optimize the parameters of individual components, such as the antenna of passive metasurfaces. Antennas are shaped over multiple simulation runs until light propagates through the structure as desired. Similar methods could be used for active metasurfaces, but instead of adjusting physical parameters, characteristics of identical antennas—such as achievable amplitude, phase, or polarization values in response to input—could be adjusted. Once an active antenna design is found, performance of the array can be optimized. Thureja et al. [80] present a mid-IR tunable metasurface using an inverse design to optimize antenna amplitude and phase in a nanoantenna array. This metasurface is based on an MOS structure integrating ITO as the active layer, allowing for beam steering and amplitude modulation. This device consists of gold patch nanoantennas with a period of 400 nm on top of a 9.5-nanometer-height HAOL layer, a 9.5 nm Al_2_O_3_ layer, a 5 nm ITO layer, and an 80 nm gold back reflector from top to bottom, optimized for 1550 nm wavelength. Simulations showed 272° of phase modulation is achieved from minimum to maximum voltage. Phase shift with constant unit amplitude allows for successful beam steering. In experiments, a phase shift of 223° was achieved with a bias voltage range of −3.6 V to 4.8 V at 1548 nm wavelength. This device was very efficient as the inverse design decreased specular reflection by around 33%, increased directivity as much as 25%, and decreased side-lobe intensity by up to 43% compared to the forward design. Inverse design methods prove to be promising for metasurface design (Figure 13).

Another application of metasurfaces is in special light modulators (SLMs). Modern SLMs maintain intensity while adjusting the phase retardation of light transmitted or reflected through each pixel, usually by using liquid crystals (LCs). LC molecules align with each other with some refractive index between that of the ordinary and extraordinary refractive indices, which can be adjusted by an applied electric field. Adding the metasurface element to an LC-SLM device to adjust the LC orientation around nanoantennas can reduce LC cell thickness to help solve the issues of crosstalk and large pixel size [77]. The authors utilized Huygens’ metasurface concept to achieve a phase shift from 0 to 2π around resonance peaks and suppression of backscattering where the induced dipole moments have equal amplitude and phase. TiO_2_ was chosen as the nanoantenna material as it has qualities of high efficiency in the visible range, negligible absorption, and a high refractive index for resonances in the LC environment. The final device comprised the 1500 nm LC layer, containing nanoantennas sandwiched between two layers of glass. Nanoantennas are 205 nm in height, with a period of 360 nm. Unit cells contain three nanoantennas per pixel. Three unit cells make a supercell, which is repeated to form the infinite gradient metasurface. Beam steering is achieved as the applied voltage is modified. After testing, an efficiency of 36% at 660 nm wavelength was achieved with a pixel size of about 1 μm and 22° FOV.

**Figure 14:**
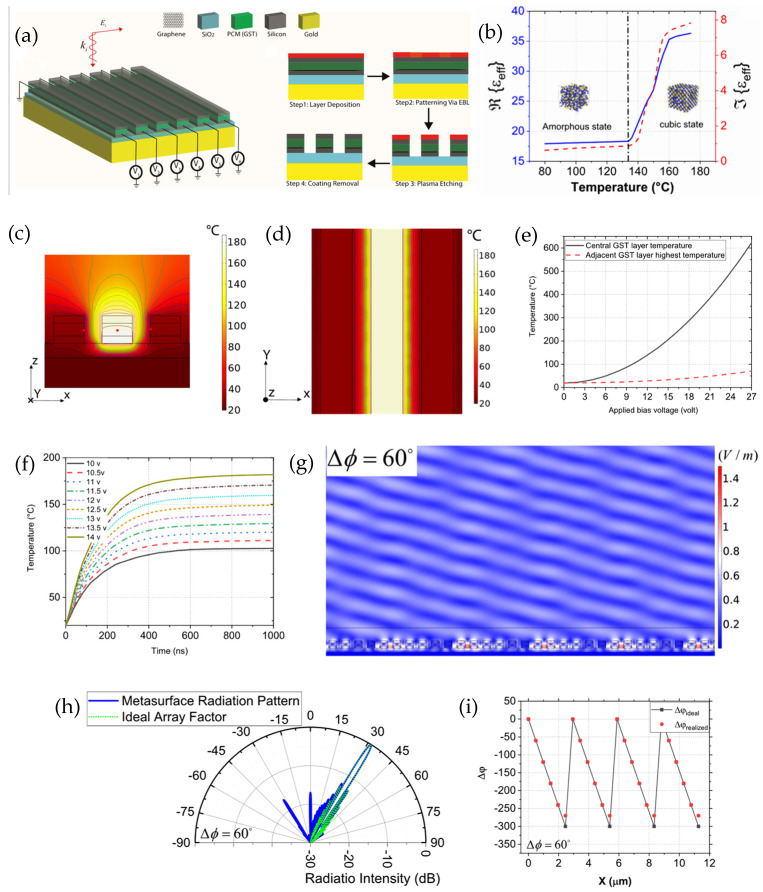
(**a**) Tunable metasurface of voltage-controlled graphene unit cells modulates x-polarized waves; a feasible fabrication process is outlined. (**b**) GST permittivity at 1.55 µm vs. temperature with real (blue) and imaginary (red, dashed) parts. (**c**) Joule heating of a unit cell under 14 V shows internal heat distribution with neighbors off (0 V); unit cells are 7 µm tall with isothermal contours. (**d**) Air heating omitted for clear internal temperature visualization. (**e**) Center GST temperature (black) and peak adjacent temperature (red, dashed) vs. bias voltage, matching red points in (**c**). (**f**) Time evolution of center GST temperature for various voltages. (**g**) Reflected-wave amplitude for a 60° progressive phase demonstrates beam steering. (**h**) Polar plot compares the metasurface’s 80-cell radiation pattern (blue) with an ideal array factor (green, dotted) at 60° phase. (**i**) Phase variation of the reflected wave of the tunable metasurface as a function of location. The black line shows the ideal phase variance, according to generalized Snell’s Law. The red dots show the realized phase variance by the proposed tunable metasurface. The plot belongs to Δ*ϕ* = 60°. (**a**–**i**) Adapted with permission from [51]. Copyright © 2020 Optical Society of America.

Thermal tuning is another mechanism to activate the metasurfaces and alter their optical properties; this can be achieved by electrothermal actuation or external thermal heating. An electric current is passed through the parts of the device to generate a localized Joule heating, causing a non-uniform temperature distribution and resulting in a physical or phase change in the material [81,82,83,84]. Thermal tuning was used by Abed et al. [51], and they present a tunable dielectric metasurface designed for fast, non-mechanical optical beam steering at the telecom wavelength of 1.55 μm, an important capability for compact, low-cost LiDAR. Each subwavelength (approximately 490 nm) unit cell comprises a germanium–antimony–telluride (GST) phase-change layer only 130 nm thick, encapsulated between low-loss silicon layers and supported by a SiO_2_ dielectric spacer over a gold reflector. Beneath the GST layer, a transparent graphene sheet functions as an electro-thermal heater. When bias voltage is applied, Joule heating raises the GST’s temperature to tune its crystallization level continuously from fully amorphous (m = 0) to fully crystalline (m = 1). Because GST is non-volatile, once a crystallization level is set, it remains stable at room temperature without sustained power. Full-wave simulations using COMSOL illustrate that the structure supports first-order Mie (magnetic dipole) resonance at 1.55 µm, producing strong amplitude and phase sensitivity to GST’s refractive index change. By carefully optimizing the geometry, the design achieves broad pulse modulation (0° to 270°) while keeping amplitude fluctuations low, favoring operation near low-m values where optical absorption is minimal. Joule heating analysis reveals that 10.5–13.6 V applied to a single graphene heater uniformly heats its GST layer to the crystallization point within approximately 500 ns, followed by approximately another 400–500 ns to complete the structural transition. This enables about a 1 μs overall tuning. Adjacent cells remain thermally isolated and reset to the amorphous state can be triggered with brief higher-voltage pulses while staying below graphene’s damage threshold of roughly 800 °C. By independently controlling each cell, the metasurface imposes a programmable phase gradient. The generalized Snell’s law (Equation (15)) predicts beam steering of the reflected wave from −65° to +65° for a range of 130° with side-lobe profiles under −10 dB. Furthermore, simulations support these results. Traditional solid-state LiDAR often relies on microelectromechanical systems (MEMS) mirrors or scanners to deflect beams. While effective, MEMS introduce moving parts, bulky and slower mechanical responses, and can face wear and vibration issues. In contrast, combining rapid, reversible GST phase transition with transparent, low-loss graphene heating, this electronically reconfigurable metasurface provides an entirely solid-state platform that performs beam steering without moving components. This approach not only complements but ultimately supersedes MEMS-based beam steering, offering a compact and energy-efficient alternative for integrated LiDAR and other dynamic nanophotonic systems such as holography and adaptive beam shaping, which paves the way for scalable, non-mechanical beam steering (Figure 14).

**Figure 15:**
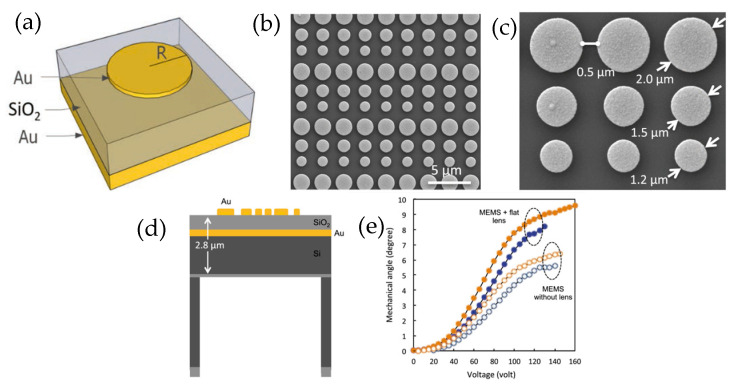
(**a**) Example unit cell of metasurface. SEM images of MEMS scanner in (**b**) broad view and (**c**) close up with measurements. (**d**) Diagram cross-section of flat lens with layers. (**e**) Angle displacement of flat lens per voltage applied. Orange represents the displacement when inner axis is actuated while using comb drives on the gimbal frame. Blue represents the displacement when outer axis is actuated using comb drives on the frame and substrate. (**a**–**e**) Adapted with permission from [91]. Copyright © 2018 The Author(s).

Mechanically tunable metasurfaces achieve dynamic control by physically changing the geometry or relative position of their components. Lateral translation or rotation of cascaded metasurface layers and microelectromechanical system (MEMS)-based actuation are some of the mechanisms used for 2D beam steering, with large tunability, precision, low power, and fast reconfiguration [85,86,87,88,89]. MEMS technologies are presented by Lyu et al. [90] as a leading contender for agile, compact beam steering. Single-axis and dual-axis MEMS mirrors can scan at kilohertz rates with deflection angles up to several tens of degrees, offering a balance between mechanical accuracy and solid-state scalability. The authors also discuss hybrid designs such as tilting metasurface mirrors that merge MEMS actuation with the optical flexibility of metasurfaces to enhance steering precision and efficiency without increasing the size. The paper ultimately identifies metasurfaces and MEMS as complementary technologies driving the evolution of solid-state LiDAR MEMS, providing a mature, manufacturable solution with proven performance, while metasurfaces offer the potential for ultimate miniaturization and photonic integration. A MEMS-integrated, tunable metasurface lens for beam steering operating at 4.6 μm was achieved by Roy et al. [91]. It consists of a flat lens stack atop an SOI wafer comprising an array of unit cells of 50-nanometer-thick disc-shaped gold resonators, a 400-nanometer-thick silicon dioxide layer, a 200-nanometer-thick gold film, a 2-micrometer-thick top device layer, and a 200 nm buried-oxide layer from top to bottom. This device is mounted on a 1 mm by 1 mm 2D electrostatic comb drive scanner with around 1 kHz resonance. Spacing the gold discs with various radii produces an adjustable phase profile that allows for ±9° tilt (Figure 15).

The focusing characteristic of this flat lens was simulated using Lumerical’s FDTD solver. They determined the focal length to be 5 mm, and the FWHM of the focal beam to be 22.8 μm. Experimentally, the FWHM was measured to be a similar value of 26.2 μm; this difference is possibly due to fabrication errors or a high $M^{2}$ value of 1.2. At angles up to 2.5°, the focused beam profile and full width at half-maximum is preserved. The authors propose that the device could be tilted so that incident light is collected around the design angle of 45° at higher angles of tilt to retain these parameters. The MEMS with a flat lens was able to achieve a much higher mechanical angle than the MEMS without a lens, nearly up to 10° with vs. 7° without. This device and successful testing prove the realization of active flat optical devices with metasurfaces and MEMS for uses like LiDAR.

Moitra et al. [92] present a comprehensive exploration of dielectric metasurfaces designed for dynamic optical wavefront control through Mie-resonant nanostructures. Their work emphasizes high-efficiency phase and amplitude modulation enabled by resonant displacement currents within subwavelength dielectric scatterers. The study details how spatially varying phase profiles can be engineered by tuning the electric and magnetic dipole resonances, allowing for full 2π phase coverage without relying on lossy plasmonic materials. The authors further demonstrate that all dielectric metasurfaces achieve low loss, polarization-independent performance in the visible and near-infrared regimes, qualities essential for compact beam steering and imaging systems. Experimental and simulation results confirm precise control over transmitted wavefronts, including deflection, focusing, and holographic projection. The paper also discusses fabrication combability with CMOS processes, reinforcing their scalability for integrated photonic applications. From a LiDAR metasurface perspective, this work is significant because it establishes foundational physical mechanisms and material considerations for realizing efficient, solid-state beam-steering devices. By leveraging Huygens’ metasurfaces and spectral overlap between electric and magnetic resonances, the approach minimizes backscattering and enhances transmission, directly contributing to the development of phase gradient metasurfaces for optical phased arrays. These principles underpin emerging LiDAR architectures that seek to replace mechanical scanning with nanophotonics control of directionality and phase. Consequently, the paper provides one of the key theoretical and experimental baselines for metasurface-based LiDAR sensors discussed here, particularly regarding the trade-off between efficiency, bandwidth, and fabrication tolerance in high-index dielectric platforms such as TiO_2_ and Si. The summary of tunable metasurfaces discussed in this section can be reviewed in Table 2. 

## 5. Metasurface-Based LiDAR Sensors

**Figure 16:**
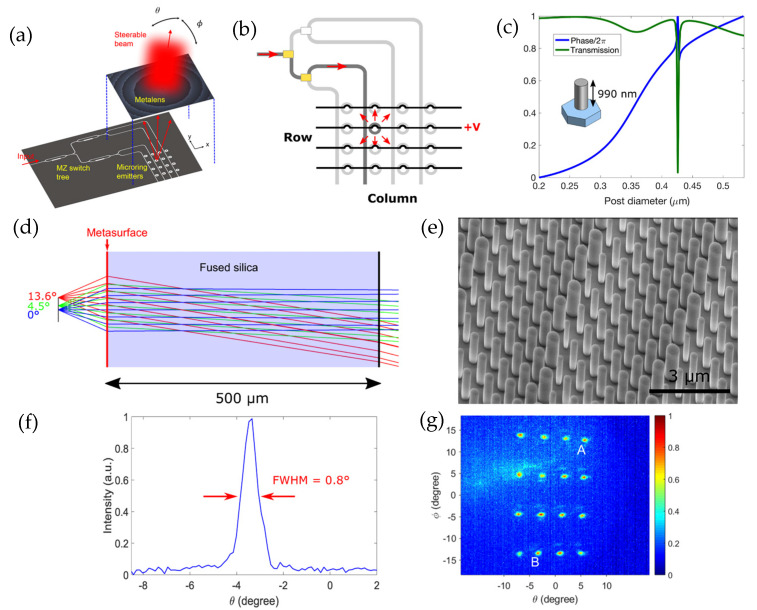
(**a**) Two-dimensional beam-steering device consisting of active-silicon-photonics microring emitter array and metalens. (**b**) Microrings are activated using microheaters by applying voltage. (**c**) Design of metasurface by using rigorous coupled-mode analysis (RCWA). (**d**) Ray tracing of point emitters. (**e**) Scanning electron microscopy image of metalens. (**f**) FWHM of divergence angle in θ direction. (**g**) Far-field angular distribution overlaps 16 different steering directions. (**a**–**g**) Adapted with permission from [93]. Copyright © Optical Society of America.

In recent years, Chang et al. [93] achieved 2D beam steering by combining a metalens with an active silicon photonics microring emitter array. A schematic diagram of the beam-steering device is shown in Figure 16a. The platform uses a single-wavelength laser source, distributing the light among different microring emitters using the tree of Mach–Zehnder (MZ) as a switch. The 4 × 4 microring emitters are placed near the waveguides, and each row of microrings is powered by microheaters using applied voltage, as shown in Figure 16b.

The metalenses used here are designed for a focal length of 90 μm and for an FOV of 13.6° by rigorous coupled-mode analysis (RCWA), shown in Figure 16c,d. The height of the silicon post arranged in a hexagonal lattice is 990 nm, and the post diameter varies from 200 nm to 534 nm to produce a phase shift from 0 to 2π. Then, the fabricated metalenses, with the SEM image shown in Figure 16e, are placed on top of the silicon platform to convert the light coming from the different emitters into different directions in the far field. The gap between the metalens and the silicon chip is 90 μm, which corresponds to the focal length of the metalens. The electrical power is consumed by MZ switches and microheaters and microrings are 83 mW. Overlapping the far-field distribution coming from each 4 × 4 emitter results in 16 different steering directions. An FOV of 12.4° × 26.8° has been achieved experimentally with a beam divergence of 0.9°, as shown in Figure 16f. Each direction is spaced by 4.1° × 8.9°, as shown in Figure 16g. With single-wavelength operation, this 2D beam-steering device shows potential for compact, efficient, and scalable LiDAR sensors.

**Figure 17:**
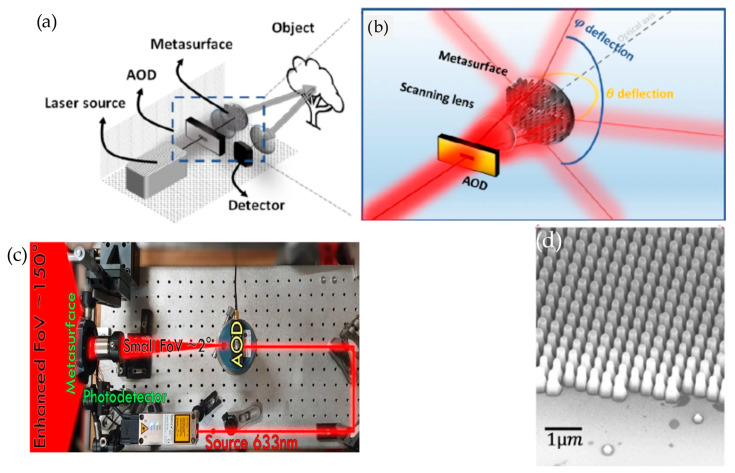
(**a**) Schematic of LiDAR system working principle of pulsed laser source for TOF detection to AOD, lens, and metasurface. (**b**) Diagram of AOD, lens, and system for light projection. (**c**) System test setup with small-FOV AOD and large-FOV metasurface with photodetector to collect reflected light. (**d**) SEM bird’s eye view image of metasurface posts. (**a**–**d**) Adapted with permission from [47]. Copyright © 2022 The Author(s).

Martins et al. [47] showed that a large FOV (150°) is achieved by using large-area metasurfaces acting as a deflection-angle amplifier from a low-FOV source. The schematic diagram of the setup is shown in Figure 17a. A single-pulse laser source is directed towards an acousto-optic deflector (AOD) that offers light scanning with a low FOV (2°). A scanning lens is used after the AOD to scan the laser spot on the metasurface at different radial r and azimuthal $\theta_{MS}$ positions. Applying voltage to the AOD can redirect the beam within 2° × 2° FOV; therefore, it can sweep the beam across the metasurface to cover a scanning range between −75° and 75°. Then, the reflected beam from the object is collected using a fast detector.

Metasurfaces used in this work are designed to accumulate the propagation phase in pillars with a controllable Effective Refractive Index (ERI). It transmits the light with spatially increasing momentum with respect to radial dimension ***r***. Radial phase retardation can be expressed as follows [47]:

(20) $$\frac{\partial \phi }{\partial r}=-k_{0}\frac{r}{r_{max}}$$

where $k_{0}$ is the free space momentum; ϕ is the local-phase retardation. At the peripheral points ${\pm r}_{max}$, the phase retardation ϕ is $\mp \frac{\pi r_{max}}{\lambda }$, and at the center of the metasurface, it is 0. Using Equation (20), the deflected angles at both axes (*θ* and *φ*) can be derived from the generalized Snell’s law and expressed as

(21) $$\left\{\begin{array}{l} k_{0}\sin\theta_{t}\sin\varphi_{t}=-k_{0}\frac{r}{r_{max}}\cos\theta_{MS}\\ \;\;\;\;\;\;\;\;\;\;\;\;\;\;\;\;\;\;\;\;\;\;\;\;\;\;\;\;\;\;\;\;\;\;\;\;\;\;\;\;\;\;\;\;\;\;\;\;\;\;\;\;\;\;\;\;\;\;\;\;,\\ k_{0}\sin\theta_{t}\cos\varphi_{t}=-k_{0}\frac{r}{r_{max}}\sin\theta_{MS} \end{array}\right.$$

One-dimensional and two-dimensional imaging is achieved using the system shown in Figure 17c. For 3D imaging, an additional FOV dimension is added by implementing a second orthogonally oriented AOD. An FOV over 150° has been reported and shown by the authors.

**Figure 18:**
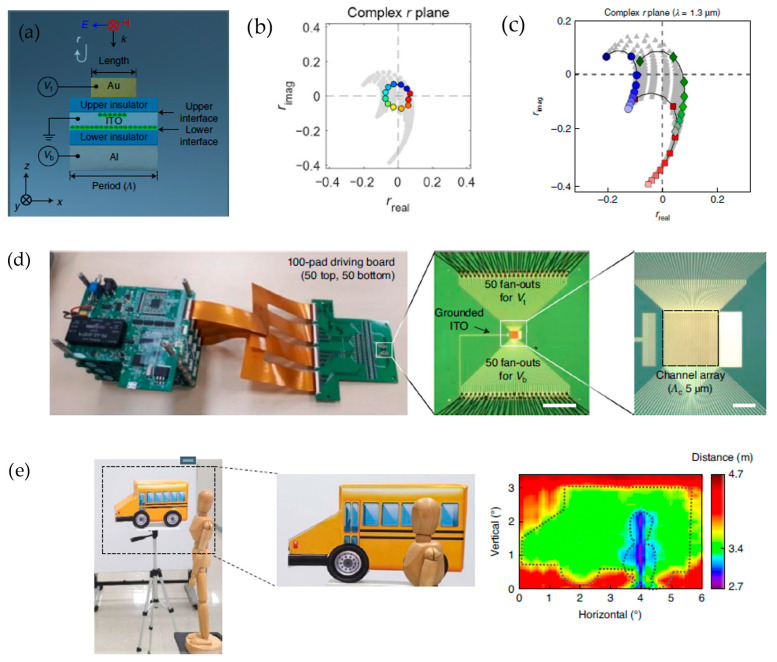
(**a**) A nanoresonator with an aluminum mirror. (**b**) Complex r-plane covering all four quadrants and providing a continuous 0–360° phase change. (**c**) Variation in the top input voltage while holding the bottom input voltage at the fixed values of −6.4 V, 3.2 V, and 6.4 V. This produces three graphs, each of the same “hook” shape. (**d**) Driving electronics independently controlled 50 top and 50 bottom electrodes, forming a 50-channel array of 550 total nanoresonators within a 250 × 250 μm^2^ active area. (**e**) The reconstructed information depth achieved up to 4.7 m from the screen behind the model car. (**a**–**d**) Adapted with permission from [95]. Copyright © 2020 The Author(s), under exclusive license to Springer Nature Limited.

Park et al. [94] demonstrated a 360° continuous sweep of an electrically tunable metasurface array, which can detect at a range of 4.7 m. A nanoresonator with an aluminum mirror is used as a one-port resonator, and its reflection coefficient, r, was obtained (Figure 18a). The manipulation of the nanoresonator is influenced by the input voltages for the top and bottom of the nanoresonator’s structure. The ranges for the voltages were between −4 V and 4 V for the top input and −6.4 V and 6.4 V for the bottom input. This particular set of values for the input voltages was selected in order for the resultant r values of the complex r-plane to cover all four quadrants and provide a continuous 0–360° phase change (Figure 18b). The top input voltage is varied while holding the bottom input voltage at the fixed values of −6.4 V, 3.2 V, and 6.4 V. This produced three graphs, each of the same “hook” shape (Figure 18c). Upon further examination of the first graph of the 360° circular phase shift on the complex r-plane, we can see the total set of hooks that can exist for the set of ranges for the input voltages. This implies that a 360° phase sweep is only possible as long as the 2D areal coverage of the voltage set permits a circular traversal of all four quadrants. After validating full 360° phase control, the authors implemented the concept of the metasurface nanoresonator into a metaphonic spatial light modulator (SLM) consisting of individually addressable nanoresonator channels (each with top and bottom electrical gates). The driving electronics independently controlled 50 top and 50 bottom electrodes, forming a 50-channel array of 550 total nanoresonators within a 250 × 250 μm^2^ active area (Figure 18d). This enabled programmable control of the reflected phase and amplitude across the surface. By applying specific voltage patterns, the study demonstrated beam splitting and steering through engineered phase and amplitude gratings. Using a sawtooth phase profile, the device achieved dynamic beam steering with a side-suppression ratio of +2.7 dB, a deflection efficiency of 34–48%, and an estimated modulation speed of 5.4 MHz, all in a solid-state, non-mechanical platform. To showcase a practical use case, the metasurface was integrated into a solid-state LiDAR prototype operating at 1.56 µm. The device performed 3D time-of-flight scanning of an emulated street scene containing a model car and a wooden human figure. The reconstructed information depth achieved was up to 4.7 m from the screen behind the model car (Figure 18e). This demonstration confirmed that the two-control-parameter metasurface can function as a high-speed, compact, beam-steering element for LiDAR and other optical systems, combining full 360° phase modulation with mechanical robustness and semiconductor integrability.

**Figure 19:**
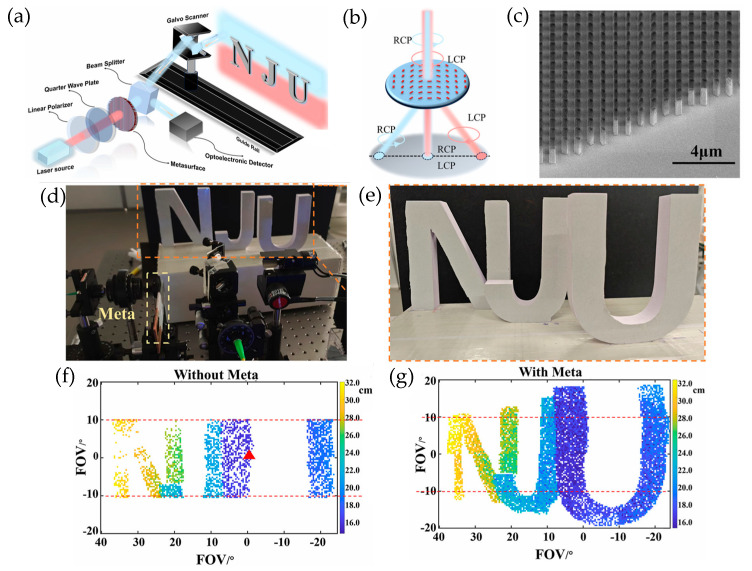
(**a**) Metasurface used as a beam splitter. (**b**) Rotating mechanical mirror. (**c**) Experimental setup showing the three letters to be imaged and camera capturing equipment. (**d**) True color image of captured frame for measured FOV. (**e**) Experimental imaging scene of staggered lettered arrangement “NJU.” (**f**) First case of limited (no metasurface modulation) horizontal FOV of −20° to 40° and vertical FOV of −10° and 10°. (**g**) Improved case with added metasurface modulation of FOV of 64° horizontally and FOV of 40°. (**a**–**g**) Adapted with permission from [95].

Precision distance measurement and velocity measurement can be utilized by using metasurfaces as polarization beam splitters to enhance the system’s FOV (Figure 19a). Chen et al. [95] demonstrate a novel FMCW LiDAR by using a mechanical rotating mirror (Figure 19b) and metasurface enlarging the FOV from 64° × 20° to 64° × 40°. At the same time, millimeter-level precision was achieved in distance measurement, and 9 mm/s velocity precision was achieved using velocimetry (Figure 19c). Three model letters were set up on a camera capture system to test the modulated metasurface’s extended capability (Figure 19d,e). A two-axis galvanometer was biased with the voltage range of −6 V and +10 V horizontally and −2.5 V and +2.5V vertically, without metasurface modulation, corresponding to a horizontal FOV of −20° to 40° and vertical FOVs of −10° and 10°. Figure 19f shows that the image of three letters, when using only a galvanometer, cannot be seen clearly. By adding the metasurface polarization beam splitter, an FOV of 64° horizontally and FOV of 40° were achieved (Figure 19g), exceeding the mechanical scanning limitation of the galvanometer and doubling the FOV.

## 6. Summary and Perspective

To sum up, in this review, first, LiDAR signal principles in a hard Lambertian object are presented in two different schemes when the receiver area is filled by the field of view and when it is not filled by the field of view. Based on sensing principles, the types of LiDAR are discussed as pulsed time of flight, amplitude-modulated continuous-wave, and frequency-modulated continuous-wave. Different scanning platforms, such as mechanical LiDAR, micromechanical LiDAR, and solid-state LiDAR, are presented. Metasurfaces—two-dimensional flat optics—are introduced as an alternative scanning platform for LiDAR.

Metasurfaces offer planar platforms and have a compact form; therefore, it is easier to integrate them into other optoelectronic and photonic systems [32,33,34,35]. Metasurfaces can be fabricated in CMOS-compatible facilities by using deep-ultraviolet (DUV) lithography, reactive ion etching (RIE), and atomic layer deposition (ALD). This enables large-area, high-volume, and cost-effective metasurface production [39,40,41,42,43,44]. In this paper, the physical meaning and mathematical representation of phase shift, deflection angle, and field of view are discussed extensively. Passive metasurfaces that result in wide-angle FOVs are discussed [48,49]. In active metasurfaces, electrical gating, thermal, optical, and mechanical actuation as tuning mechanisms are reviewed for beam steering [50,51,52,91]. Two-dimensional and three-dimensional image formation by metasurface-based LiDAR is shown [47,93,96].

In the near future, to achieve compact, high-speed, and low-cost LiDAR, III-V laser diodes, a metasurface beam-steering platform, and a high-speed detector should be integrated on a single chip. While silicon metasurfaces and germanium detectors can be monolithically grown on the silicon substrate, III-V laser diode integration can be challenging. Integration techniques of III-V lasers on silicon platforms, such as monolithic and heterogeneous integration, have been extensively researched to achieve efficient coupling between the active region of the laser structure and the silicon material [97]. Flip-chip bonding of III-V lasers such as vertical cavity surface emitters (VCSELs) onto silicon platforms can provide high output power, low threshold current, and stable operation [98,99,100,101]. Overall, integrating silicon metasurfaces with III-V lasers on a silicon platform is a promising solution for future solid-state LiDAR sensing applications. Monolithic integration of metasurfaces on a photonic-crystal surface-emitting laser (PCSEL) was demonstrated by Hsu et al. [102] to realize a chip-scale light projector for depth perception. This work shows an important pathway for integrating metasurfaces on chips for LiDAR.

## Figures and Tables

**Table 1:** Summary of passive metasurfaces.

Metasurface Device	Application	Material	Phase Shift	FOV	Transmission/Efficiency	Reference/Year
Cylindrical rods with varying radii	Wide FOV	Silicon/SiO_2_	1.62π	60.4°	83% transmission	[46]/2025
Cylindrical meta-atoms	Wide-angle beam projector	Silicon/glass	2π	120°	90% transmission	[49]/2025
Metasurface diffractive optical elements	Wide FOV	Amorphous silicon/fused silica	2π	120° × 120°	96% transmission	[48]/2020
Grating-based guided-mode resonance filter	Echo-band-pass filtering	Silicon/SiO_2_	π and 2π	N.A.	1% transmission	[66]/2024
Dammann gratings	Beam splitting and combining	Amorphous silicon/SiO_2_	π	N.A.	90% efficiency/ 97% uniformity	[68]/2023
Computationally optimized metasurface	Beam shaping	Hydrogenated amorphous silicon/silica	2π	360°	N.A.	[58]/2024
V-shaped metasurfaces	Beam shaping with anomalous reflection	Au/MgF_2_	3π/4	45.5° ^1^	80% transmission	[69]/2012

^1^ Deflection angle from normal.

**Table 2:** Summary of tunable metasurfaces.

Metasurface Type	Tuning Mechanism	Material	Phase Shift	FOV	Reference/Year
Independently addressable nanoantennas	Electro-optic effect	ITO/Al_2_O_3_/HAOL/Au	270°	22° ^2^	[70]/2020
Plasmonic patch antenna	Electrical gating	ITO/Al_2_O_3_ /MIM	250°	66.5° ^2^	[71]/2016
Metasurface reflectarray antenna	Electrical gating	MOS/Al_2_O_3_/ITO	184°	40°	[72]/2016
Tamm plasmon polariton	Electrical bias	Au/Graphene /ITO	208°	45° × 270°	[78]/2023
Geometric phase metasurface of nanobricks	Electrical bias	Sb_2_S_3_	342° ^1^	101.6°	[79]/2024
Plasmonic nanoribbons	Electrical bias	Graphene	360° ^1^	72°	[73]/2020
Multiple-quantum-well metasurface	Electrical gating/ Stark effect	GaAs/AlGaAs	70°	19.3°	[50]/2019
Metasurface array	Electrically tunable/ Inverse design	MOS/HAOL /ITO/Au	223°	70°	[80]/2020
Spatial light modulator	Electrical gating/ LC reorientation	TiO_2_	90°	22°	[77]/2019
Dielectric metasurface	Electro-thermal	Graphene/GST	270°	130°	[51]/2020
Metasurface array	Electrically tunable/ Inverse design	Au/Graphene	310°	30°	[90]/2021
Disc-shaped nanoresonators	Electro-mechanical	Au/SiO2	252° ^1^	18°	[91]/2018
Mie-resonant nanostructures	Electrical gating/ LC reorientation	TiO2/ITO	309° ^1^	22°	[92]/2023

^1^ Phase shifts are given in terms of π in the original paper and converted into degrees for the table. ^2^ Beam deflection angles from normal to horizontal in one dimension.

## Data Availability

No new data were created or analyzed in this study. Data sharing is not applicable to this article. Data availability may vary and can be requested from the original articles that were reviewed in this review paper.
